# Determinants Present in the Receptor Carboxy Tail Are Responsible for Differences in Subtype-Specific Coupling of *β*-Adrenergic Receptors to Phosphoinositide 3-Kinase

**DOI:** 10.1155/2009/959168

**Published:** 2009-02-05

**Authors:** Julie Simard, Matthieu Boucher, Rachel Massé, Terence E. Hébert, Guy Rousseau

**Affiliations:** ^1^Département de Pharmacologie, Université de Montréal, Montréal, PQ, Canada H3C 3J7; ^2^Centre de Biomédecine, Hôpital du Sacré-Cœur de Montréal, 5400 boulevard Gouin Ouest, Montréal, PQ, Canada H4J 1C5; ^3^Department of Pharmacology and Therapeutics, McGill University, Montreal, PQ, Canada H3G 1Y6

## Abstract

An agonist-occupied *β*_2_-adrenergic receptor (*β*_2_-AR) recruits G protein receptor kinase-2 (GRK2) which is recruited to the membrane. Thus, the physical proximity of activated *β*_2_-AR and PI-3K allows the activation of the latter. In contrast, it has been observed that the *β*_1_-AR is unable to activate the PI-3K/Akt pathway. We hypothesized that the difference might be due to molecular determinants present in the carboxy termini of the two *β*-AR subtypes. Using transiently transfected HEK 293 cells expressing either *β*_1_- or *β*_2_-AR, we also observed that in presence of an agonist, *β*_2_-AR, but not *β*_1_-AR, is able to activate the PI-3K/Akt pathway. Switching the seventh transmembrane domain and the carboxy tail between the two receptors reverses this phenotype; that is, *β*_1_ × *β*_2_-AR can activate the PI-3K/Akt pathway whereas *β*_2_ × *β*_1_-AR cannot. Pretreatment with pertussis toxin abolished the activation of PI-3K by *β*_2_- or *β*_1_ × *β*_2_-AR stimulation. Ligand-mediated internalization of the *β*_2_-AR induced by a 15-minute stimulation with agonist was abolished in the presence of a dominant negative of PI-3K or following pertussis toxin pretreatment. These results indicate that the subtype-specific differences in the coupling to PI-3K/Akt pathway are due to molecular determinants present in the carboxy tail of the receptor and further that *β*_2_-AR activates PI-3K via a pertussis toxin-sensitive mechanism.

## 1. Introduction

*β*_2_-AR activation induces antiapoptotic effects in cardiomyocytes mediated by stimulation of the PI-3K pathway [1]. The proposed mechanism by which this activation occurs is dependent on G protein-coupled receptor kinases (GRKs). Under basal conditions, GRK2 forms a complex with PI-3K in the cytosol [2]. When *β*_2_-AR is occupied by an agonist, GRK2 is translocated to the membrane by a G*β* *γ* subunit-dependent mechanism and subsequently recruits PI-3K from the cytosol to the membrane [2]. The proximity of the PI-3K and the stimulated receptor induces the activation of the enzyme. In contrast, *β*_1_-AR has proapoptotic activity in cardiomyocytes due to stimulation of PKA or CAM KII [3]. Indeed, it has been observed that stimulation of *β*_1_-AR with an agonist induces apoptosis and this can be prevented in presence of inhibitors of either PKA or CAM-KII [4].

Interestingly it has been shown that stimulated *β*_1_-AR can also recruit GRK to the membrane. The interaction of either *β*-AR subtype with GRKs is mainly via the intracellular loops and carboxy tail of the receptor [5]. When activated, GRK induces the phosphorylation of certain serine/threonine residues in the carboxy tail of both *β*-AR subtypes resulting in functional uncoupling of the receptor from their primary signalling pathways. GRK2 phosphorylation also favours subsequent interaction with *β*-arrestin. This interaction with *β*-arrestin further desensitizes the receptor and is subsequently involved in receptor endocytosis [6, 7].

As GRK interacts with molecular determinants in carboxy tail of the receptor, we hypothesized that differences in the coupling of the *β*_1_- and *β*_2_-AR with PI-3K may be due to determinants located in this portion of the receptor. This study was designed to determine the respective efficiencies of *β*_1_-AR and *β*_2_-AR to stimulate the PI-3K pathway and to test the above hypothesis.

## 2. Material and Methods

### 2.1. DNA Constructions, Cell Transfection, and Culture

Murine *β*_1_-AR and human *β*_2_-AR subcloned into pcDNA3 were used in this study. Two chimeric receptors consisting of the *β*_1_-AR with the seventh transmembrane domain and the carboxyl-terminal tail of the *β*_2_-AR and the reciprocal *β*_2_-AR with the seventh transmembrane domain and the carboxyl-terminal tail of the *β*_1_-AR were constructed as follows. A restriction site for Hpa I was created by polymerase chain reaction (PCR) at position 2070 in the *β*_1_-AR. The new restriction site in the *β*_1_-AR was created with three primers. Two primers were used for the hybridation with the receptor sequence. These primers contained 21 base pairs and had, respectively, CGCCTCAGAAGCCATAGAGCC and TCGTGTGCACAGTGTGGGCCA sequences. The third primer was utilized to introduce the restriction site. This primer which contained 24 base pairs had the following sequence: GGTGGAAAGCGTTAACCACGTTGG. The mutated *β*_1_-AR and the *β*_2_-AR wt were double digested with Hpa I and Xho I. The result of this digestion is two fragments of 6540 bp and 486 bp for the mutated *β*_1_-AR and two fragments 5998 bp and 1362 bp for the *β*_2_-AR wt. The appropriate restriction fragments (containing the seventh transmembrane domain and the carboxy-terminal portion of the receptor) were isolated, exchanged for their counterparts, and religated. Positive clones were selected by enzymatic digestion and confirmed by sequencing.

The *β*-AR wild type (wt), chimeric receptors, dominant-negative PI-3 kinase (p85ΔPI-3K), and/or carboxy-terminal domain of GRK2 (ct-GRK2) were transiently transfected in human embryonic kidney (HEK 293) cells using the calcium phosphate precipitation method. We performed all experiments at 48 hours posttransfection, that is, at maximal receptor expression determined by ligand binding. Cells were starved overnight 24 hours before the experiments in a medium without fetal bovine serum. HEK 293 cells were maintained in DMEM supplemented with 10% fetal bovine serum, 100 U/mL penicillin and streptomycin, 1 mM glutamine, 0.25 *μ*g/mL fungizone in an atmosphere of 95% air/5% CO_2_ at 37°C. On the day of the experiment, cells were treated with 1 *μ*M isoproterenol for the indicated times and fractionated for the cytosolic or membrane compartments. In some experiments, cells were pretreated with pertussis toxin (0.1 *μ*g/mL; Sigma), 18 hours before stimulation with isoproterenol.

### 2.2. Preparation of Cytosolic and Membrane Fractions

Cells were washed three times with 10 mL of phosphate-buffered saline at 4°C and mechanically detached in 1 mL of ice-cold buffer containing 5 mM Tris-HCl, pH 7.4, 2 mM EDTA, 5 *μ*g/mL leupeptin, 5 *μ*g/mL soybean trypsin inhibitor, and 10 *μ*g/mL benzamidine. Cells were then lysed with a sonicator (3 bursts of 10 seconds at max speed), and the lysates were centrifuged at 1000 × g for 5 minutes at 4°C. The supernatant was centrifuged at 45 000 × g for 20 minutes and was considered as the cytosolic preparation. Protein content was assessed using the Bradford method (Bio-Rad). The pelleted membranes were resuspended in 250 *μ*L of a solubilization buffer (buffer A) containing 50 mM Tris pH 7.5, 20 mM *β*-glycerophosphate, 20 mM NaF, 5 mM EDTA, 10 mM EGTA, 1 mM Na_3_VO_4_, 10 mM benzamidine, 0.5 mM PMSF, 10 *μ*g/mL leupeptin, 5 mM DTT, 1 *μ*M microcystin LR, and 1% Triton X-100; and solubilized for 2 hours at 4°C. Then the membranes were centrifuged at 10 000 × g for 15 minutes. The protein content was assessed using the Lowry method (Bio-Rad).

### 2.3. Radioligand Binding Assay

Radioligand binding assays were conducted essentially as described previously [8] with ~5 *μ*g of membrane proteins in a total volume of 0.5 mL containing 250 pM [^125^I]CYP in the presence or absence of 10 *μ*M alprenolol to define nonspecific binding. The binding reactions were incubated at room temperature for 90 minutes and terminated by rapid filtration with ice-cold 25 mM Tris-HCl, pH 7.4, over Whatman GF/C glass fiber filters preincubed for ≥30 minutes in a buffer containing 25 mM Tris-HCl, pH 7.4, 0.1% bovine serum albumin, and 0.3% polyethylenimine.

### 2.4. Western Blotting

Western blotting was conducted as described previously [9]. Briefly, aliquots of 50–75 *μ*g of cytosolic or membrane protein preparations were subjected to 10% denaturing polyacrylamide gel electrophoresis as previously described. Transfer was performed with a Trans-Blot SD Semi-dry transfer cell (Bio-Rad) on Protran nitrocellulose membrane (Mandel, Montréal, QC, Canada). Protein transfer efficiency was assessed using Ponceau S staining. Membranes were blocked using 5% nonfat dry milk in TBS-T (10 mM Tris (pH 7.4), 150 mM NaCl, and 0.05% Tween 20) and membranes were incubated at 4°C overnight with primary antibody (anti-Phospho-Akt (Ser 473) and anti-Akt from Cell Signaling Technology, (Missisauga, Canada) or anti-PI-3K, anti-*β*_1_- or anti-*β*_2_-AR from Santa-Cruz (Calif, USA) diluted 1:1000 in 5% nonfat dry milk into TBS-T. Subsequently, membranes were washed and incubated for 45 minutes at room temperature with the secondary antibody (diluted 1:5000 in 5% nonfat dry milk into TBS-T) conjugated to horseradish peroxidase. Membranes were washed and exposed to scientific imaging film (Perkin Elmer Life Sciences, ON) or quantified using a Kodak ImageStation 440CF using enhanced chemiluminescence reagent (Perkin Elmer Life Sciences). Band intensities were analyzed using Kodak 1D v.3.5.5 Scientific Imaging Software.

### 2.5. Immunohistochemistry and Receptor Internalization

Sequestration of *β*-AR was observed by immunolocalisation. After agonist treatment, cells were washed with PBS and fixed with 3% paraformaldehyde for 15 minutes. After these washes, nonspecific sites were blocked with 0.2% BSA and 0.15% Triton x-100 (blocking solution) for 10 minutes. Primary antibody (*β*_1_- and *β*_2_-AR from Santa-Cruz), prepared in the blocking solution (1:200), was added for 30 minutes at room temperature. After another series of washes, secondary antibody (antirabbit, Santa-Cruz,) also prepared in the blocking solution (1:500) was added for 30 minutes. After a final series of washes, slides were mounted and viewed using a Leica epi-illumination microscope.

### 2.6. PI-3K Activity

PI-3K activity was measured as previously described [10]. Briefly, 250–375 *μ*g of cytosolic and membrane proteins were precipitated with anti-phosphotyrosine antibody conjuged to biotin (1:50, Santa-Cruz, Calif, USA) overnight at 4°C. The immune complex was pelleted (with streptavidin beads) and washed three times with lysis buffer and twice with phosphate-buffered saline buffer containing 0.1 mM Na_3_VO_4_. The immune pellet was then suspended in activation buffer (35 mM ATP, 0.2 mM adenosine, 30 mM MgCl_2_, 10 mg/mL L-*α*-phosphatidylinositol, and 20 *μ*Ci [*γ* ^32^ *P*]-ATP; (Amersham Pharmacia Biotech, Baie-d'Urfé, Canada) and incubated at room temperature for 20 minutes. The reaction was stopped with the addition of 100 *μ*L HCl 1 M and 200 *μ*L of chloroform:methanol (1:1). The aqueous phase was then discarded. Eighty *μ*L of HCl:methanol (1:1) were then added before discarding the aqueous phase. Twenty *μ*L of the organic phase containing ^32^P-phosphatidylinositol were resolved by thin layer chromatography on K6 Silica Gel plates (Whatman, Clifton, NJ, USA) in a solvent system containing chloroform:methanol:ammonium hydroxide (45:35:10). Plates were exposed to film for three to five days (−80°C).

### 2.7. Statistical Analysis

Results are expressed as mean ± SEM and were evaluated using analysis of variance adapted for factorial experimental design. Orthogonalization was performed when necessary [11]. *P* < .05 was considered significant.

## 3. Results

### 3.1. Expression of *β*-AR Subtypes and p85ΔPI-3K in HEK 293 Cells

HEK 293 cells were transiently transfected with cDNAs encoding for *β*_1_-AR Wt, *β*_2_-AR Wt, *β*_1_ × *β*_2_-AR, or *β*_2_ × *β*_1_-AR and in some case, p85ΔPI-3K. Forty-eight hours after transfection *β*-AR expression levels were approximately 500 fmol/mg of proteins (compared to 10–20 fmol/mg in untransfected cells). Expression of p85ΔPI-3K determined by western blot was increased 3.24 times as compared to wild-type cells (data not shown, *n* = 3).

### 3.2. Stimulation of *β*_2_-AR but not *β*_1_-AR Induces Activation of PI-3K/Akt Pathway

**Figure 1:**
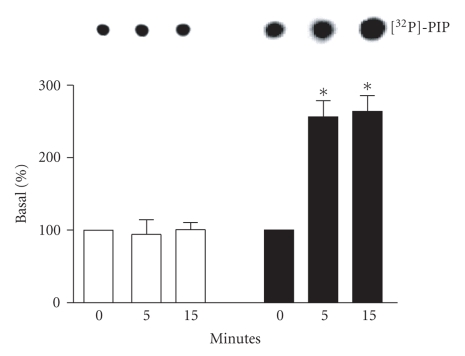
*PI-3K activity with *β*-AR stimulation*. HEK 293 cells were transfected with *β*_1_-AR or *β*_2_-AR. Forty-eight hours after transfection, cells were stimulated with 1 *μ*M isoproterenol for the indicated times. PI-3K activity was determined by the level of [^32^P]-PI produced by the stimulation of *β*_1_-AR (□) or *β*_2_-AR (■) expressing cells. Top panel is a representative autoradiograph of TLC separation (*n* = 4−5). Data from these experiments is quantitated in the bottom panel as described in Section 2 . *P < .05 versus 0 minute.

PI-3K activation by the *β*_1_- or *β*_2_-AR was measured by in vitro phosphorylation of L-*α*-phosphatidylinositol. Transfected cells were stimulated with isoproterenol 1 *μ*M for 0, 5, or 15 minutes at 37°C. Stimulation of *β*_2_-AR- for 5 or 15 minutes induced a significant augmentation in PI-3K activity compared to basal conditions (nonstimulated; Figure 1). In contrast, *β*_1_-AR stimulation had no effect on PI-3K activity. Thus, the stimulation of *β*_2_-AR but not *β*_1_-AR by isoproterenol induces activation of PI-3K in our model. We suspect that the apparent high basal level of PI-3K activation observed in our cell line may be due to the phosphotyrosine antibody used to immunoprecipitate the activated PI-3K. Using this antibody, we immunoprecipitate other PI-3K subtypes as well as phosphotyrosine proteins that although not activated by *β*-AR may still contribute to the basal level of activation.

**Figure 2:**
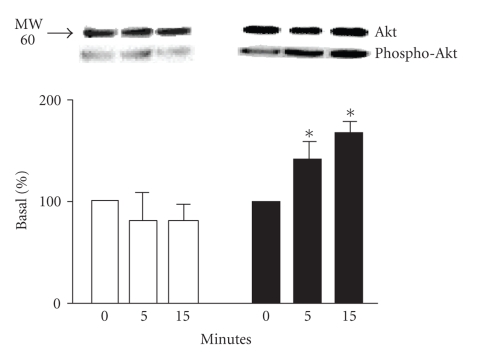
*Akt activation following *β*-AR stimulation*. HEK 293 cells were transfected with *β*_1_-AR or *β*_2_-AR. Forty-eight hours after the transfection, cells were stimulated with 1 *μ*M isoproterenol for the indicated times. Akt activation status was determined by immunoblot for the phosphorylated form of Akt compared to the total amount of Akt after stimulation of *β*_1_-AR (□) or *β*_2_-AR (■) expressing cells. Top panel is a representative immunoblot of the experiments (*n* = 4−5). Data from these experiments is quantitated in the bottom panel as described in Section 2 . *P < .05 versus 0 minute.

To confirm differences between stimulation of the two *β*-AR subtypes on PI-3K activity, we used a measure of Akt (a downstream PI-3K effector) activation. Akt activation, as determined by the phosphorylation status of Serine 473, was significantly increased after *β*_2_-AR-stimulation for 5 and 15 minutes as compared to the basal state (Figure 2). Stimulation of *β*_1_-AR did not modify the phosphorylation status of Akt as compared to control, confirming that *β*_1_-AR activation cannot stimulate PI3-kinase/Akt pathway activation. Stimulation of untransfected HEK293 with isoproterenol did not result in any significant activation of Akt (data not shown).

### 3.3. Stimulation of Either *β*_1_- or *β*_2_-AR Induces PI-3K Recruitment to the Plasma Membrane

**Figure 3:**
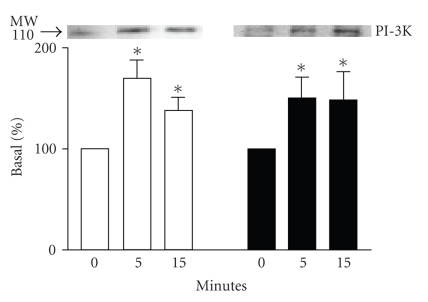
*PI-3K Partitioning of PI-3K to the membrane*. Forty-eight hours after transfection, cells were stimulated with 1 *μ*M isoproterenol for the indicated times. Membranes were isolated, solubilized, and approximately 100 *μ*g of membrane proteins were separated on a 10% SDS-PAGE gel. Membrane-associated PI-3K levels increased with cells expressing *β*_1_-AR (□) or *β*_2_-AR (■). Top panel is a representative immunoblot of the experiments (*n* = 3−4). Data from these experiments is quantitated in the bottom panel as described in Section 2 . *P < .05 versus 0 minute.

To determine whether *β*_1_-AR can recruit PI-3K to the plasma membrane, immunolocalisation of PI-3K was performed using Western blotting with anti-P110*γ* antibody. Transiently transfected cells with *β*_1_- or *β*_2_-AR were stimulated by isoproterenol 1 *μ*M for 0, 5, or 15 minutes at 37°C. Both *β*_1_- and *β*_2_-AR stimulation results in recruitment of PI-3K to the particulate fraction (Figure 3). Compared with control (i.e., nonstimulated cells), the presence of PI-3K was significantly increased (*P* < .05) in membrane fractions by agonist stimulation of either *β*_1_-AR or *β*_2_-AR for 5 or 15 minutes. No significant difference was detected between 5 and 15 minutes of stimulation. Thus, either subtype of *β*-AR can recruit PI-3K to the membrane after agonist stimulation but only the *β*_2_-AR results in PI-3K activation.

### 3.4. Recruitment of PI-3K to the Plasma Membrane is G*β* *γ* Subunit-Dependent

**Figure 4:**
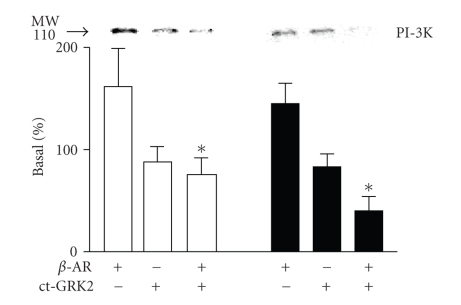
*GRK2 carboxy-terminal domain reduced PI-3K recruitment to membrane fractions in response to *β*-AR stimulation*. HEK 293 cells were cotransfected with ct-GRK-2 and *β*-AR subtypes. Forty-eight hours after the transfection, cells were stimulated with 1 *μ*M isoproterenol for 5 minutes. PI3-kinase recruitment to particulate fractions decreased with cells expressing either *β*_1_-AR (□) or *β*_2_-AR (■) in presence of cotransfected ct-GRK2. Top panel is a representative immunoblot of the experiments (*n* = 4–6). Data from these experiments is quantitated in the bottom panel as described in Section 2. *P < .05 versus 0 minute.

It has been shown that PI-3K and GRK2 form a cytosolic complex [2]. To determine whether the mechanism of PI-3K recruitment to the plasma membrane was G*β* *γ* subunit-dependent, *β*_1_- or *β*_2_-AR was transiently cotransfected with the carboxyl-terminal portion (ct) of GRK2 (a sequestering agent for G*β* *γ*) in HEK 293 cells. Cells cotransfected with *β*_1_- or *β*_2_-AR and ct-GRK2 were stimulated with 1 *μ*M isoproterenol for 5 minutes. ct-GRK2 when transfected alone had no effect on PI-3K activity. Stimulation of the *β*_2_-AR for 5 minutes with isoproterenol in cells cotransfected with ct-GRK2 resulted in a significantly decreased (*P* < .001) PI-3K recruitment to the particulate fraction (Figure 4) as compared to stimulation of the *β*_2_-AR expressed alone. Similar results were obtained when cells expressing the *β*_1_-AR were stimulated for 5 minutes with the agonist, that is, the presence of ct-GRK2 significantly decreased (*P* < .001) PI-3K recruitment to membranes (Figure 4). Thus, PI-3K recruitment to the plasma membrane by either *β*_1_- or *β*_2_-AR stimulation is G*β* *γ* subunit-dependent although only *β*_2_-AR-dependent recruitment results in subsequent PI-3K activation.

### 3.5. PI-3K/Akt Pathway Activation Following Stimulation of Chimeric *β*_1_ × *β*_2_-AR and *β*_2_ × *β*_1_-AR

**Figure 5:**
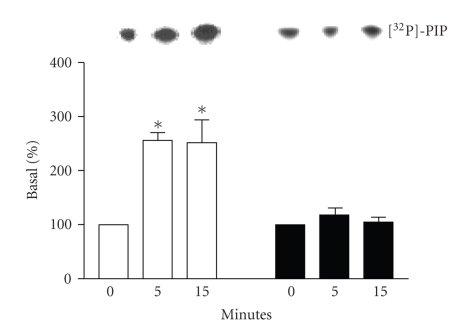
*PI-3K activity with chimeric *β*-AR stimulation*. HEK 293 cells were transfected with *β*_1_ × *β*_2_-AR or *β*_2_ × *β*_1_-AR. Forty-eight hours after transfection, cells were stimulated with 1 *μ*M isoproterenol for the indicated times. PI-3K activity was determined by the level of [^32^ *P*]-PI produced by the stimulation of *β*_1_ × *β*_2_-AR (□) or *β*_2_ × *β*_1_-AR (■) expressing cells. Top panel is a representative autoradiograph of the TLC separation (*n* = 4−5). Data from these experiments is quantitated in the bottom panel as described in Section 2. *P < .05 versus 0 minute.

To determine the importance of receptor-specific determinants in the *β*-AR carboxy tail for PI-3K activation, we constructed two chimeric receptors which consisted of the *β*_1_-AR with the proximal seventh transmembrane domain and carboxy-terminal tail of the *β*_2_-AR (*β*_1_ × *β*_2_-AR) as well as the reciprocal receptor which consisted of *β*_2_-AR with the carboxy-terminal tail of the *β*_1_-AR (*β*_2_ × *β*_1_-AR). When the *β*_1_ × *β*_2_-AR was stimulated by 1 *μ*M isoproterenol for 5 or 15 minutes, PI3-kinase activity was significantly increased (Figure 5; *P* < .05). On the other hand, agonist stimulation of the *β*_2_ × *β*_1_-AR for 5 or 15 minutes did not result in increased PI3-kinase activity. These results suggest that the carboxy-terminal tail of the *β*_2_-AR contains molecular determinants which are required for PI3-kinase activation.

**Figure 6:**
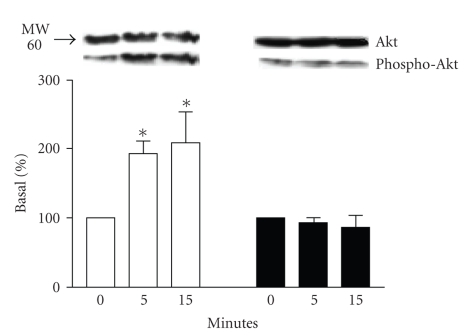
*Akt activity with chimeric *β*-AR stimulation*. HEK 293 cells were transfected with *β*_1_ × *β*_2_-AR or *β*_2_ × *β*_1_-AR. Forty-eight hours after the transfection, cells were stimulated with 1 *μ*M isoproterenol for the indicated times. Akt activity was determined by measuring levels of phosphorylated Akt compared with total Akt produced by the stimulation of *β*_1_ × *β*_2_-AR (□) or *β*_2_ × *β*_1_-AR (■) expressing cells. Upper panel is a representative immunoblot of the experiments (*n* = 6−7). Data from these experiments is quantitated in the bottom panel as described in Section 2. *P < .05 versus 0 minute.

To confirm results obtained for the PI-3K activation mediated by the two chimeric *β*-ARs, we again determined the activation status of Akt. Chimeric receptors were stimulated with 1 *μ*M isoproterenol for 0, 5, or 15 minutes at 37°C. Akt phosphorylation was significantly increased (*P* < .05) in HEK 293 cells expressing *β*_1_ × *β*_2_-AR (Figure 6). Stimulation of *β*_2_ × *β*_1_-AR by isoproterenol had no effect on Akt phosphorylation as compared to basal conditions. Thus chimeric *β*_1_ × *β*_2_-AR increased Akt phosphorylation and confirmed that the carboxy-terminal tail of the *β*_2_-AR contained molecular determinants necessary and sufficient for PI-3K activation.

### 3.6. Effect of Pertussis Toxin Treatment on *β*-AR Stimulation of PI3-K Activity

**Figure 7:**
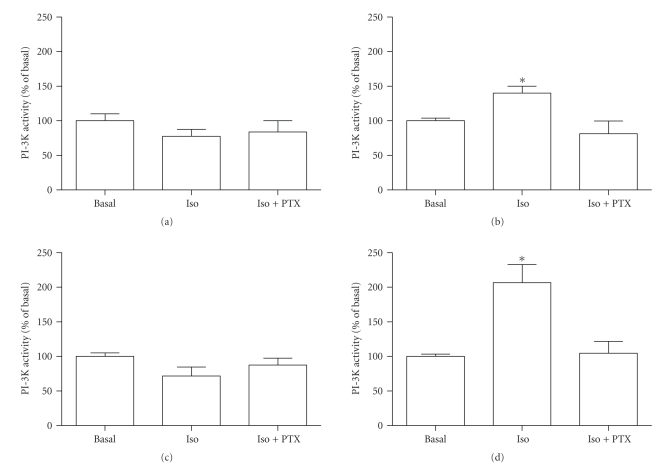
*Pertussis toxin effects on *β*-AR-mediated PI-3K activation*. Twenty-six hours after transfection, cells were incubated with 0.1 *μ*g/mL pertussis toxin for 18 hours. Cells were stimulated for 5 minutes with 1 *μ*M isoproterenol prior to processing for TLC as described in Figure 1 and Section 2. PI-3K activity was determined by the level of [^32^ *P*]-PI produced by the stimulation of *β*_1_-AR (a), *β*_2_-AR (b), *β*_2_ × *β*_1_-AR (c), and *β*_1_ × *β*_2_-AR expressing cells. (*n* = 3−4; *P < .05 versus basal and PTX-Iso conditions).

It has been proposed that stimulation of PI-3K by *β*_2_-AR is pertussis toxin sensitive [1, 12]. To determine the effect of pertussis toxin on *β*-AR induced stimulation of PI-3K, we incubated the cells with PTX for 18 hours followed by a stimulation with 1 *μ*M isoproterenol for 15 minutes. Our results indicate that, in presence of PTX, no activation of PI-3K was observed with *β*_2_-AR stimulation (Figure 7(b)). In presence of the chimeric receptors, PTX treatment abolished the activation of PI-3K observed with the chimeric *β*_1_ × *β*_2_-AR (Figure 7(d)). Consistent with our earlier data, neither the *β*_1_-AR (Figure 7(a)) nor the *β*_2_ × *β*_1_-AR (Figure 7(c)) was sensitive to PTX pretreatment. These results indicate that stimulation of PI-3K by *β*_2_-AR is pertussis toxin sensitive.

### 3.7. Involvement of PI-3K Activation on *β*-AR Sequestration

**Figure 8:**
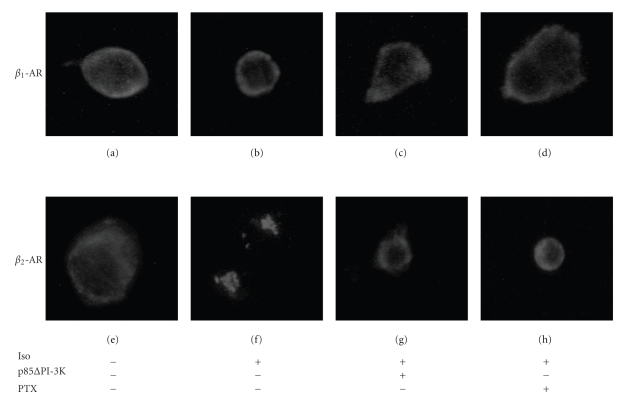
*p85ΔPI-3K reduced agonist-induced *β*_2_-AR sequestration*. HEK 293 cells were transfected with p85ΔPI-3K and the two *β*-AR subtypes. Cells were stimulated with 1 *μ*M isoproterenol for 15 minutes (b) and (f). Stimulation of *β*_2_-AR-transfected cells induced a cluster distribution which is characteristic of receptor internalization (f). This sequestration was abolished in presence of either cotransfected p85ΔPI-3K (g) or PTX pretreatment (h). No significant *β*_1_-AR sequestration was observed under the different conditions used in these experiments (a)–(d). Representative images of several experiments (*n* = 8–15) are shown.

Cells expressing *β*_1_-AR were treated with isoproterenol for 15 minutes. After stimulation, very little *β*_1_-AR was observed in the interior compartments of treated cells and no significant difference was observed in presence of either cotransfected p85ΔPI-3K or PTX pretreatment (Figures 8(a)  to 8(d)). In contrast, we observed a significant internalization of *β*_2_-AR after a similar period of agonist stimulation as shown by clustered distribution of the receptor (Figure 8(f); outline of the cell is poorly defined in this particular condition). *β*_2_-AR internalization was inhibited in the presence of either cotransfected p85ΔPI-3K or PTX pretreatment (Figures 8(g) and 8(h)). These results confimed that functional PI-3K is important for the sequestration of *β*_2_-AR and attests to the effectiveness of our p85ΔPI-3K cotransfection or PTX pretreatment.

## 4. Discussion

The results of the current study demonstrate that stimulation of *β*_2_-AR, but not *β*_1_-AR, induces PI-3K activation by a pertussis toxin-sensitive mechanism. This subtype-dependent activation has been reinforced by the fact that Akt, a downstream PI-3K effector, was also selectively activated by *β*_2_-AR stimulation. However, both receptor subtypes can recruit the PI-3K to the plasma membrane via a G*β* *γ* subunit-dependent mechanism. Naga Prasad et al. [2] had previously demonstrated that PI-3K and GRK2 formed a cytosolic complex and the recruitment of the enzyme to the plasma membrane was facilitated by G*β* *γ* subunits in an agonist-dependent manner. Our results confirmed that PI-3K is recruited to the plasma membrane via a G*β* *γ* subunit-dependent mechanism. GRK-2 is important for PI-3K recruitment to the plasma membrane, thus we thought this might suggest that the carboxyl-terminal domain of the receptor is important for the PI-3K activation because the carboxyl-terminal domain of the *β*-AR contains sites phosphorylated by GRK2. Furthermore, Shiina et al. [13] had demonstrated that the carboxyl-terminal domain and the third cytosolic loop are the regions mostly responsible for the difference in internalization behavior between both *β*-AR subtypes.

To determine the potential contribution of the carboxyl-terminal domains of the *β*_1_- and *β*_2_-AR in the PI-3K activation, chimeric *β*_1_- and *β*_2_-AR in which the seventh transmembrane domain and the carboxyl-terminal domain have been exchanged were constructed. We observed that the chimeric *β*_1_ × *β*_2_-AR could activate PI-3K in contrast to the wild type *β*_1_-AR. Reciprocally, *β*_2_-AR lost its ability to activate PI-3K when its carboxy-terminal domain was exchanged for that of the *β*_1_-AR. This result demonstrated that the *β*_2_-AR carboxyl-terminal domain contains important molecular determinants for PI-3K activation.

The present study confirms PI-3K activation by *β*_2_-AR stimulation as observed in other studies [2, 12]. However the kinetics of PI-3K activation previously reported for *β*_2_-AR stimulation was different to that observed in the present study. PI-3K was rapidly activated following *β*_2_-AR stimulation and returned to basal levels after 10 minutes. In our study, we observed a significant PI-3K activation with a 5-minute *β*_2_-AR stimulation that was maintained after 15 minutes. The difference in activation patterns may be due to the agonist concentration used. In the previous study [2], 10 *μ*M isoproterenol was used which is 10 times higher than the concentration used here. Higher concentration of agonist may induce more rapid PI-3K recruitment to the membrane and thus activation of the enzyme but may also more rapidly stimulate mechanisms which terminate these signals as well.

Pretreatment with pertussis toxin results in a loss of PI-3K activation by *β*_2_-AR stimulation confirming results obtained in other studies [1, 12]. The new information afforded by the present study is that the carboxy-terminal portion of the receptor is important for the interaction between the receptor, Gi heterotrimers (as stimulation depends on both the G*α* and G*β* *γ* subunits), and PI-3K. Indeed, the chimeric receptor *β*_1_ × *β*_2_-AR can activate the PI-3K by a pertussis toxin sensitive mechanism whereas the chimeric receptor *β*_2_ × *β*_1_-AR is unable to activate PI-3K. However, other regions of the receptor may also be important for full activation or regulation of the response by the desensitization machinery.

Our results demonstrate that isoproterenol-stimulated *β*_1_-AR (for up to 15 minutes) cannot activate PI-3K. These results contrast with those reported in a previous study in which activation of PI-3K was observed after stimulation of *β*_1_-AR transfected into HEK 293 cells [2]. Several possibilities can explain the discrepancy between both studies. First, 1 *μ*M isoproterenol although sufficient to induce submaximal adenylyl cyclase activity [8] cannot induce PI-3K activation. Secondly, in our study we use transiently transfected cells that express approximately 500 fmol receptor/mg of membrane proteins, which is closer to physiological levels than seen with the study of Naga Prasad et al. [2]. Also, it is clear that the interplay between the signalling and internalization machinery is different for the two receptors even though they can interact with many of the same proteins. This study provides another such example that both *β*_1_-AR and *β*_2_-AR lead to PI-3K and GRK2 recruitment; both the signalling and desensitization outcomes for these events are markedly different. Some studies have also shown that *β*_1_-AR, in contrast to the *β*_2_-AR, cannot activate Gi [14] while others suggest that it can [15]. It is possible that other cell- and tissue-specific factors might regulate these events as well.

In this study, we observed that *β*_2_-AR internalization is inhibited in the presence of a dominant negative PI-3K, p85ΔPI-3K, results that are similar to previous studies [2, 16]. We also observed that *β*_2_-AR sequestration can be abolished with pertussis toxin pretreatment. In contrast to *β*_2_-AR, we observed that the *β*_1_-AR sequestration is minimal with 15 minutes of stimulation. This is similar to the 10% sequestration observed in *β*_1_-AR expressing cells obtained by Suzuki et al. [17]. Since phosphorylated lipids generated by PI-3K are critical for the receptor internalization dynamics [18, 19], it is plausible that the weak sequestration observed for the *β*_1_-AR might be due to the weak PI-3K activation induced by the stimulation of this receptor subtype. Results obtained with the pertussis pretreatment and p85ΔPI-3K also suggest that activated PI-3K is important for receptor sequestration.

In conclusion, we observed that PI-3K activation is *β*-AR subtype specific and the difference is due to molecular determinants present in the carboxy-terminal tail of the receptors. PI-3K activation is pertussis toxin sensitive and is necessary for the sequestration of the *β*_2_-AR.
